# Implications of High Sensitivity Troponin Levels After Lung Transplantation

**DOI:** 10.3389/ti.2024.12724

**Published:** 2024-04-11

**Authors:** Eduard Rodenas-Alesina, Adriana Luk, John Gajasan, Anhar Alhussaini, Genevieve Martel, Cyril Serrick, Karen McRae, Chris Overgaard, Marcelo Cypel, Lianne Singer, Jussi Tikkanen, Shaf Keshavjee, Lorenzo Del Sorbo

**Affiliations:** ^1^ Division of Cardiology, Department of Medicine, University of Toronto, Toronto, ON, Canada; ^2^ Ted Rogers Centre for Heart Research, Peter Munk Cardiac Centre, University Health Network, Toronto, ON, Canada; ^3^ Interdepartmental Division of Critical Care Medicine, University Health Network, Toronto, ON, Canada; ^4^ Perfusion Services, University Health Network, Toronto, ON, Canada; ^5^ Department of Anesthesia and Pain Management, University Health Network, Toronto, ON, Canada; ^6^ Southlake Regional Healthcare Centre, Newmarket, ON, Canada; ^7^ Division of Thoracic Surgery, Faculty of Surgery, University of Toronto, Toronto, ON, Canada; ^8^ Toronto Lung Transplant Program, Ajmera Transplant Center, University Health Network, Toronto, ON, Canada; ^9^ Division of Respirology, Faculty of Medicine, University of Toronto, Toronto, ON, Canada

**Keywords:** lung transplant, troponin, primary graft dysfunction, mechanical ventilation, arrhythmia

## Abstract

Trends in high-sensitivity cardiac troponin I (hs-cTnI) after lung transplant (LT) and its clinical value are not well stablished. This study aimed to determine kinetics of hs-cTnI after LT, factors impacting hs-cTnI and clinical outcomes. LT recipients from 2015 to 2017 at Toronto General Hospital were included. Hs-cTnI levels were collected at 0–24 h, 24–48 h and 48–72 h after LT. The primary outcome was invasive mechanical ventilation (IMV) >3 days. 206 patients received a LT (median age 58, 35.4% women; 79.6% double LT). All patients but one fulfilled the criteria for postoperative myocardial infarction (median peak hs-cTnI = 4,820 ng/mL). Peak hs-cTnI correlated with right ventricular dysfunction, >1 red blood cell transfusions, bilateral LT, use of EVLP, kidney function at admission and time on CPB or VA-ECMO. IMV>3 days occurred in 91 (44.2%) patients, and peak hs-cTnI was higher in these patients (3,823 vs. 6,429 ng/mL, *p* < 0.001 after adjustment). Peak hs-cTnI was higher among patients with had atrial arrhythmias or died during admission. No patients underwent revascularization. In summary, peak hs-TnI is determined by recipient comorbidities and perioperative factors, and not by coronary artery disease. Hs-cTnI captures patients at higher risk for prolonged IMV, atrial arrhythmias and in-hospital death.

## Graphical Abstract

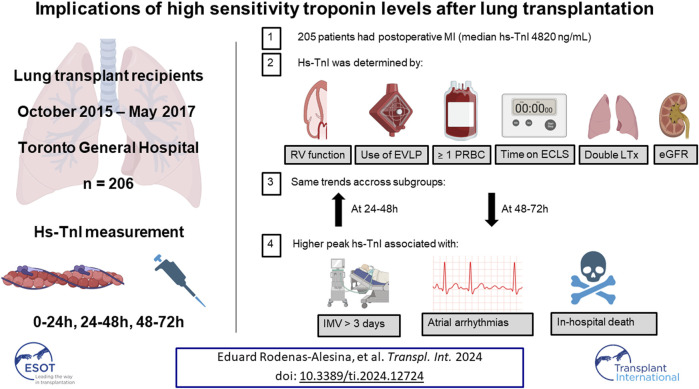

## Introduction

The use of high sensitivity cardiac troponin I (hs-cTnI) measurement after cardiac surgery is well stablished, and is associated with higher risk of death, prolonged invasive mechanical ventilation (IMV), and prolonged length of stay. In cardiac surgery, there are multiple factors involved in postoperative hs-cTnI levels, such as ischemia-reperfusion injury, cannulation and manipulation, type of cardioplegia used, inflammation and elevation of filling pressures, with direct damage to the coronary artery tree being rare [1]. Despite its recognized predictive value, routinary measurement on hs-TnI immediately after surgery is not useful in certain settings, such as in heart transplantation [2].

In non-cardiac surgery, hs-cTnI is commonly used to rule out perioperative myocardial injury following non-cardiac surgery (MINS), which has been associated with higher mortality, atrial arrhythmias and admissions for heart failure [3–5]. The mechanisms leading to hs-cTnI rise are not fully elucidated, but seem to be related as well with perioperative conditions and baseline characteristics, and consistently associated with worse outcomes irrespective of the presence of ischemic symptoms or ECG changes [6], with a dose-graded response based on degree of post-operative hs-cTnI elevation [7]. With the development newer hs-cTnI assays, the incidence of perioperative MINS will likely increase as it is now recommended to screen high-risk patients postoperatively [7].

The use of hs-cTnI in the lung transplant (LTx) population has not been well studied. During LTx, hemodynamic stability and direct cardiac damage may confer a different value to hs-cTnI to that of non-cardiac surgery, but there is paucity of data regarding the elevation of hs-cTnI in the early postoperative course of LTx recipients, limited by sample size and lack of serial measurements [8, 9]. With the new hs-cTnI assays replacing the old ones, it remains crucial to examine the normal trend in hs-cTnI in LTx to guide decision-making.

This study sought was to evaluate the levels and trend of hs-cTnI during the first 72 h after LTx, factors impacting on hs-cTnI levels and the prognostic value of hs-cTnI in the intensive care unit (ICU) setting.

## Materials and Methods

### Patient Population

Consecutive patients (≥18 years of age) who received a LTx from October 2015 to May 2017 who were admitted to Toronto General Hospital, University Health Network medical-surgical intensive care unit (MSICU) were included in this retrospective registry. The study protocol was approved by the local Research Ethics Board (CAPCR study #17-5633).

### Data Collection and Measurement

Data was collected from the electronic medical record. Pre-operative demographics, co-morbid medical illnesses, medications, and cardiac testing was recorded. As part of their lung transplant workup, patients underwent extensive cardiac testing including electrocardiography, transthoracic echocardiography, non-invasive stress testing, coronary angiography and right heart catheterization. RV dysfunction was categorized as none, mild, moderate, or severe based on the visual assessment on the echocardiography. Mild coronary artery disease (CAD) was defined as any non-obstructive coronary lesion <70% with <50% for left main disease. Any CAD more than mild was revascularized prior to listing.

Surgical interventions were performed by thoracic surgeons, along with a dedicated group of thoracic anesthesiologists, according to the Toronto Lung Transplant Program technique [10]. On a case-by-case basis, surgery was performed on cardiopulmonary bypass (CPB), veno-arterial extracorporeal membrane oxygenation (VA-ECMO) or without mechanical circulatory support (MCS). We collected the use of MCS before and after the surgery, and the length of MCS for the intraoperative period. Ischemic time and reperfusion arterial blood gas samples were also recorded.

Hs-cTnI was measured during the first 24 h, between 24 and 48 h and between 48 and 72 h after LTx on a routine basis. Peak hs-cTnI was considered the highest hs-cTnI among three measurements available. The hs-cTnI assay used at our institution was the Abbott Alinity high sensitivity troponin I assay, with a 99% upper limit of normal of 26 ng/L.

### Definition of Endpoints

The primary endpoint was prolonged IMV defined as IMV for >3 days. Secondary endpoints included in-hospital mortality, postoperative atrial arrythmias (inclusive of atrial fibrillation, atrial flutter, or atrial tachycardia), length of stay in ICU and primary graft dysfunction (PGD) 72 h post LTx, defined by current guidelines based on a P/F ratio <300 72 h after LTx with lung infiltrates on a chest X ray [11]. All patients were followed up until death or hospital discharge, whichever occurred first, and there were no patients lost to follow-up.

### Statistical Analysis

Mann-Whitney U test was used to assess differences in baseline characteristics at the time of admission, and median and interquartile range are provided. For categorical variables, proportions were compared using a chi-squared test, and counts and percentages are given.

To determine clinical factors determining peak hs-cTnI level, univariate analysis with linear regression for preoperative and surgical variables was done. Variables with missing in >25% were not used for multivariate analysis, and we performed complete case analysis. For the multivariate analysis, a backwards stepwise selection method (*p* < 0.05 for inclusion, *p* > 0.10 for exclusion) was conducted to include all relevant variables among those with a *p*-value <0.2 in univariate analysis. Collinearity within the final model was considered unacceptable if variance inflation factor was >4. The trends in hs-cTnI levels after LTx were assessed using a mixed-effects model for repeated measures, and both intercepts and slopes were compared between prespecified subgroups. Prespecified comparison groups to assess trends in postsurgical hs-cTnI trends were performed based on pre-existing non-flow limiting CAD, pulmonary hypertension, right ventricular (RV) dysfunction, use of preoperative ECMO, use of MCS during surgery, bilateral LTx, use of *ex vivo* lung perfusion system (EVLP) and number of blood transfusions required during surgery.

Peak hs-cTnI was tested as a predictor for the primary and secondary endpoints. Medians between patients with and without the endpoint were compared using U Mann Whitney test. Univariate analysis was performed using logistic regression and an optimal cutoff for each endpoint was selected using receiver-operator curve (ROC) analysis and Youden’s index. Temporal trends were compared between groups in a mixed effects model for repeated measurements. Multivariate analysis for the primary endpoint was done by including in a backwards stepwise regression all clinically relevant predictors with a *p*-value <0.2. For in-hospital mortality, as there were a relatively low number of events, bivariate analyses for *a priori* clinically relevant covariates were done. To obtain the probability of the primary endpoint based on peak hs-cTnI levels, peak hs-cTnI was modelled as a restricted cubic spline with 3 knots. A two-tailed *p*-value <0.05 was considered significant for all comparisons. Analyses were performed using Stata 15.0 for Mac (StataCorp LLC, Texas, United States).

## Results

During the study period, 206 LTx patients were included (Table 1). Median age was 58 (48–64) years and 35.4% were women. Intestitial lung disease was the most common indication for LTx (52.0%), followed by chronic obstructive pulmonary disease (20.1%), with 79.6% of patients receiving a bilateral LTx. Though 73.4% of recipients had mild CAD following coronary angiography, only 9.3% received percutaneous coronary intervention prior to listing. RV dysfunction and atrial arrhythmias were infrequent at the time of surgery. Only 1.9% of patients were bridged to LTx with VV-ECMO, and 7.3% with VA-ECMO. However, VA-ECMO was applied intraoperatively in 43.2%, and CPB in 6.8% of cases. EVLP was used in 32% of cases.

**TABLE 1:** Baseline characteristics of the cohort divided according to prolonged invasive mechanical ventilation (>3 days).

	Total (*n* = 206)	IMV for ≤3 days (*n* = 115)	IMV for >3 days (*n* = 91)	*p*-value
Age	58.2 (48.1–64.4)	59.9 (49.1–65.4)	57.8 (47.2–62.4)	0.19
Female sex	73 (35.4%)	39 (33.9%)	34 (37.4%)	0.61
BMI (kg/m^2^)	24.7 (20.3–28.7)	24.9 (20.3–28.4)	24.1 (20.5–29.0)	0.92
Hypertension	39 (18.9%)	18 (15.7%)	21 (23.1%)	0.18
Etiology				0.45
COPD	41 (20.1%)	28 (24.6%)	13 (14.4%)	
Cystic fibrosis	24 (11.8%)	12 (10.5%)	12 (13.3%)	
Pulmonary hypertension	10 (4.9%)	3 (2.6%)	7 (7.8%)	
Sarcoid	2 (1.0%)	1 (0.9%)	1 (1.1%)	
Retransplant	2 (1.0%)	1 (0.9%)	1 (1.1%)	
Interstitial lung disease	106 (52.0%)	58 (50.9%)	48 (53.3%)	
** **Other	19 (9.3%)	11 (9.6%)	8 (8.9%)	
Dyslipidemia	42 (20.4%)	19 (16.5%)	23 (25.3%)	0.12
Diabetes	40 (19.4%)	17 (14.8%)	23 (25.3%)	0.059
Non-flow limiting CAD	149 (73.4%)	83 (73.5%)	66 (73.3%)	0.98
Previous MI	4 (2.0%)	3 (2.6%)	1 (1.1%)	0.43
History of PCI	19 (9.3%)	11 (9.6%)	8 (8.9%)	0.87
History of CABG	5 (2.4%)	3 (2.6%)	2 (2.2%)	0.85
Atrial fibrillation or flutter	4 (2.0%)	2 (1.8%)	2 (2.2%)	0.82
Chronic heart failure	1 (0.5%)	0 (0.0%)	1 (1.1%)	0.26
Right ventricular function				0.51
** **Normal	149 (72.3%)	85 (73.9%)	64 (70.3%)	
** **Mild	37 (18.0%)	22 (19.1%)	15 (16.5%)	
** **Moderate	10 (4.9%)	4 (3.5%)	6 (6.6%)	
** **Severe	10 (4.9%)	4 (3.5%)	6 (6.6%)	
Chronic kidney disease	2 (1.0%)	1 (0.9%)	1 (1.1%)	0.86
Cerebrovascular accident	4 (1.9%)	1 (0.9%)	3 (3.3%)	0.21
Hemoglobin (g/L)	142.0 (128.0–152.0)	144.0 (134.0–154.0)	137.0 (123.0–150.0)	0.006
Sodium (mmol/L)	140.0 (138.0–141.0)	140.0 (138.0–141.0)	140.0 (137.0–141.0)	0.84
Potassium (mmol/L)	4.0 (3.7–4.2)	4.0 (3.8–4.1)	4.0 (3.7–4.2)	0.55
eGFR (mL/m^2^/1.73 m^2^)	94.0 (79.0–105.0)	94.0 (78.0–104.0)	93.5 (79.0–107.0)	1.00
Bridged with VV-ECMO	4 (1.9%)	0 (0.0%)	4 (4.4%)	0.023
Bridged with VA-ECMO	15 (7.3%)	3 (2.6%)	12 (13.2%)	0.004
Bilateral lung transplant	164 (79.6%)	84 (73.0%)	80 (87.9%)	0.009
Use of EVLP	66 (32.0%)	32 (27.8%)	34 (37.4%)	0.15
ECMO intraoperatively	89 (43.2%)	38 (33.0%)	51 (56.0%)	<0.001
CPB intraoperatively	14 (6.8%)	5 (4.3%)	9 (9.9%)	0.12
Time on intraoperatively MCS	0.0 (0.0–199.0)	0.0 (0.0–123.0)	70.0 (0.0–243.0)	<0.001
Reperfusion PCO_2_	0.4 (0.4–0.5)	0.5 (0.4–0.5)	0.4 (0.4–0.5)	0.070
Reperfusion O_2_	1.0 (1.0–1.0)	1.0 (1.0–1.0)	1.0 (1.0–1.0)	0.95
Number of PRBCs				<0.001
No PRBC needed	92 (45.1%)	63 (55.3%)	29 (32.2%)	
1 PRBC used	69 (33.8%)	44 (38.6%)	25 (27.8%)	
>1 PRBC used	43 (21.1%)	7 (6.1%)	36 (40.0%)	
VV-ECMO after surgery	11 (5.3%)	1 (0.9%)	10 (11.0%)	0.001
VA-ECMO after surgery	10 (4.9%)	4 (3.5%)	6 (6.6%)	0.30
Ischemic time—left lung (min)	480.5 (409.5–609.5)	477.5 (402.0–626.0)	489.5 (419.0–589.0)	0.82
Ischemic time—right lung (min)	465.0 (373.0–629.0)	459.0 (361.0–573.0)	475.0 (394.0–652.0)	0.20
Troponin at 24 h (ng/mL)	3347.0 (1863.0–5823.0)	2749.5 (1556.0–4538.0)	4386.0 (2660.0–7716.0)	<0.001
Troponin at 48 h (ng/mL)	4339.0 (2615.0–6680.0)	3696.0 (2050.0–5999.0)	5314.0 (3594.0–8371.0)	<0.001
Troponin at 72 h (ng/mL)	3235.0 (2050.0–4800.0)	2875.0 (1824.0–3995.0)	3520.0 (2492.5–6321.5)	0.003
Peak troponin (ng/mL)	4820.0 (2894.0–7331.0)	3823.0 (2392.0–5992.0)	6429.0 (3873.0–9418.0)	<0.001

IMV, invasive mechanical ventilation; BMI, body mass index; COPD, chronic obstructive pulmonary disease; CAD, coronary artery disease; MI, myocardial infarction; PCI, percutaneous coronary intervention; CABG, coronary artery bypass graft; eGFR, estimated glomerular filtration rate; VV-ECMO, veno-venous extracorporeal membrane oxygenation; VA-ECMO, veno-arterial extracorporeal membrane oxygenation; EVLP, *Ex-ViVo* Lung Perfusion; CPB, cardiopulmonary bypass; MCS, mechanical circulatory support; PRBC, packed red blood cells.

**FIGURE 1:**
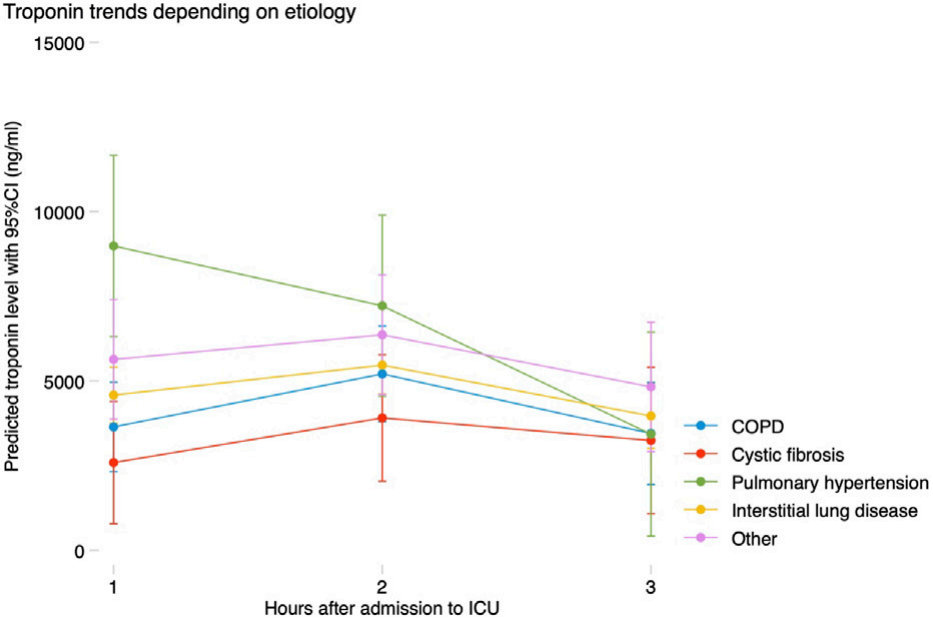
Trends in troponin according to etiology of the underlying lung disease. COPD, chronic obstructive pulmonary disease; CI, confidence interval; ICU, intensive care unit.

All patients met the criteria for MINS, and 99.5% of them even reached a hs-cTnI 10 times above the upper limit of normal, which is considered the threshold to define a coronary artery bypass graft-related MI [12]. No patients suffered from a ST elevation MI. Hs-cTnI levels at 0–24 h, 24–48 h and 48–72 h were available in 207 (100%), 185 (89.4%) and 137 (66.2%) patients, respectively. Median hs-cTnI level in the first 24 h was 3,347 (1863–5,823) ng/mL, and maximum hs-cTnI in the first 72 h was 4,820 (2,894–7,331) ng/mL. After LTx, hs-cTnI progressively increased reaching a peak between 24 and 48 h, and then decreased between 48 and 72 h. This trend was similar in all the prespecified subgroups assessed, with higher baseline levels in patients with pulmonary hypertension, who experienced a steeper decline after LTx (Figure 1).

**FIGURE 2:**
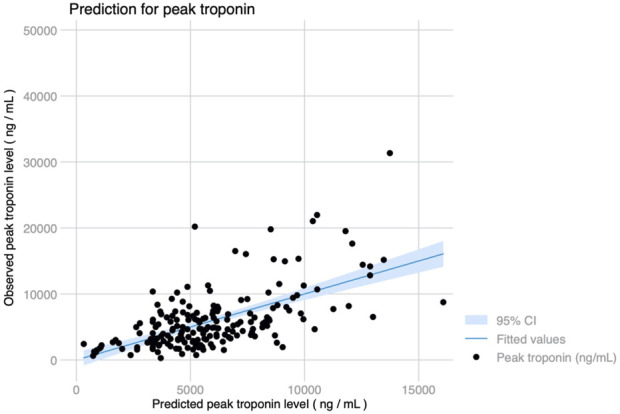
Observed and predicted peak troponin levels using a 7-variable model including RV dysfunction, hypertension, eGFR, bilateral lung transplant, use of EVLP, length of intraoperative MCS and transfusion requirements (Pearson’s r = 0.604, *p* < 0.001).

Peak hs-cTnI within the first 72 h was higher in patients with dyslipidemia, hypertension, previous heart failure, pulmonary hypertension, poor RV function, chronic kidney disease or mild CAD. Patients supported with either VV or VA-ECMO preoperatively, those receiving a bilateral LTx or patients in which EVLP was used had greater hs-cTnI levels postoperatively. The use of MCS (either CPB or VA-ECMO) during surgery and the time on support also were strongly associated with hs-cTnI rise, as well as the number of transfusions required. Independent predictors for peak hs-cTnI levels are shown in Table 2, and were severe RV dysfunction, eGFR at the time of LTx, use of EVLP, receiving a bilateral LTx, prolonged time on MCS intraoperatively and receiving >1 red blood cell transfusion. Predicted hs-cTnI levels using this model had a strong correlation with the observed peak hs-cTnI (Pearson’s r = 0.604, *p* < 0.001) (Figure 2).

**TABLE 2:** Effect on peak troponin within the initial 72 h after lung transplant of each predictor in univariate and multivariate linear regression analysis.

	Univariate		Multivariate	
	Peak troponin	*p*-value	Peak troponin	*p*-value
Hypertension	1,316	0.190	1,277	0.074
Dyslipidemia	2,245	0.021	—	—
Chronic kidney disease	19,063	0.001	—	—
Chronic heart failure	9,187	0.104	—	—
Mild coronary artery disease	1,407	0.123	—	—
Pulmonary hypertension	3,086	<0.001	—	—
Severe RV dysfunction	4,129	0.023	3,612	0.011
eGFR (per mL/min/1.73 m^2^)	−55	0.007	−40	0.005
Bridged with VV-ECMO	4,167	0.143	—	—
Bridged with VA-ECMO	−1,977	0.191	—	—
Bilateral lung transplant	3,869	<0.001	3,288	<0.001
Use of EVLP	1,996	0.018	1,528	0.010
VA-ECMO during surgery	2,672	0.001	—	—
Time on MCS (per min)	16	<0.001	9	<0.001
>1 PRBC used	4,640	<0.001	1,827	0.011

The multivariate model was chosen based on a backwards stepwise regression.

RV, right ventricular; eGFR, estimated glomerular filtration rate; VV-ECMO, veno-venous extracorporeal membrane oxygenation; VA-ECMO, veno-arterial extracorporeal membrane oxygenation; CPB, cardiopulmonary bypass; MCS, mechanical circulatory support; PRBC, packed red blood cells.

**FIGURE 3:**
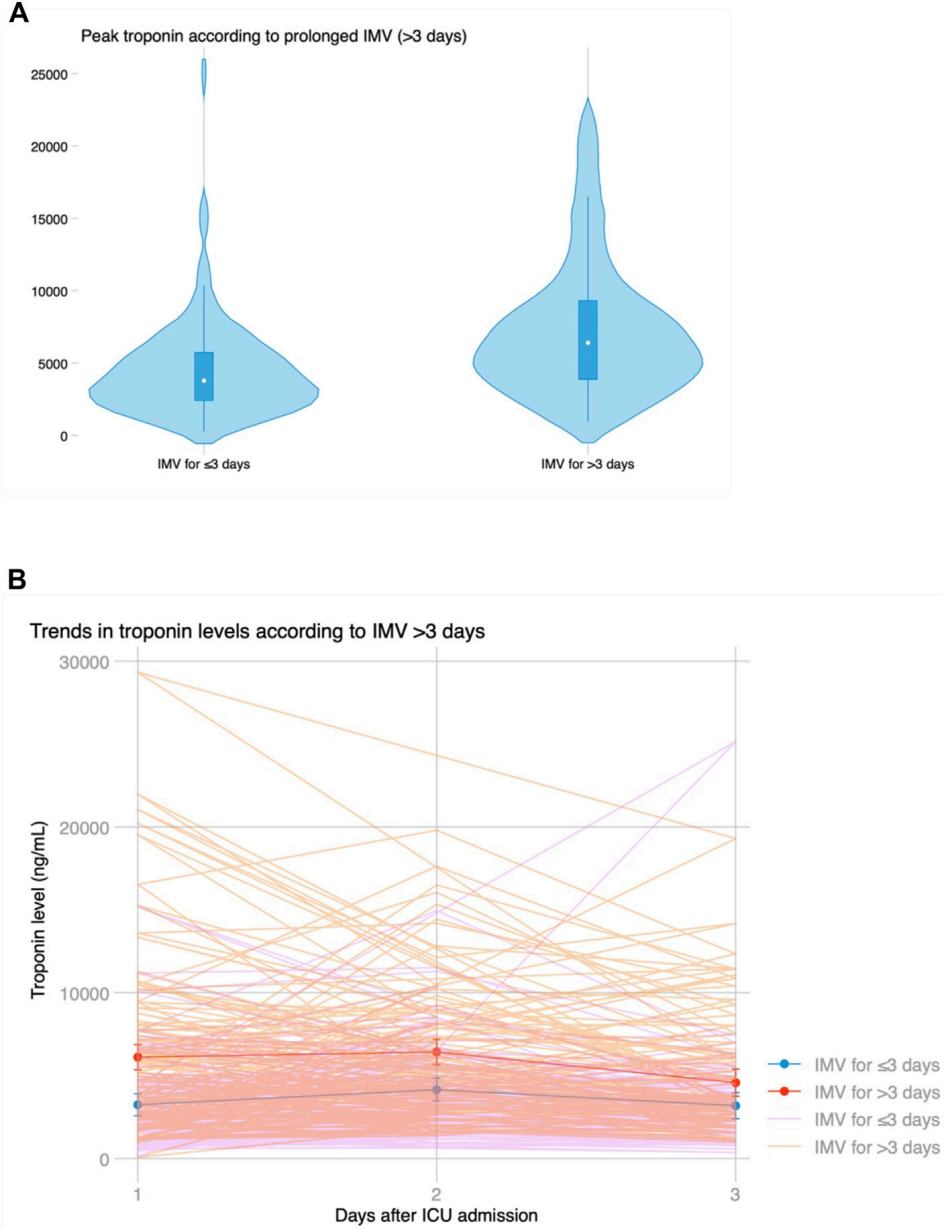
**(A)** Violin plot showing peak troponin based on prolonged invasive mechanical ventilation for >3 days. **(B)** Temporal trends in troponin levels after LT based on prolonged ventilation, showing significantly higher initial troponin levels but similar kinetics and progressive decline on the third day in both groups.

**FIGURE 4:**
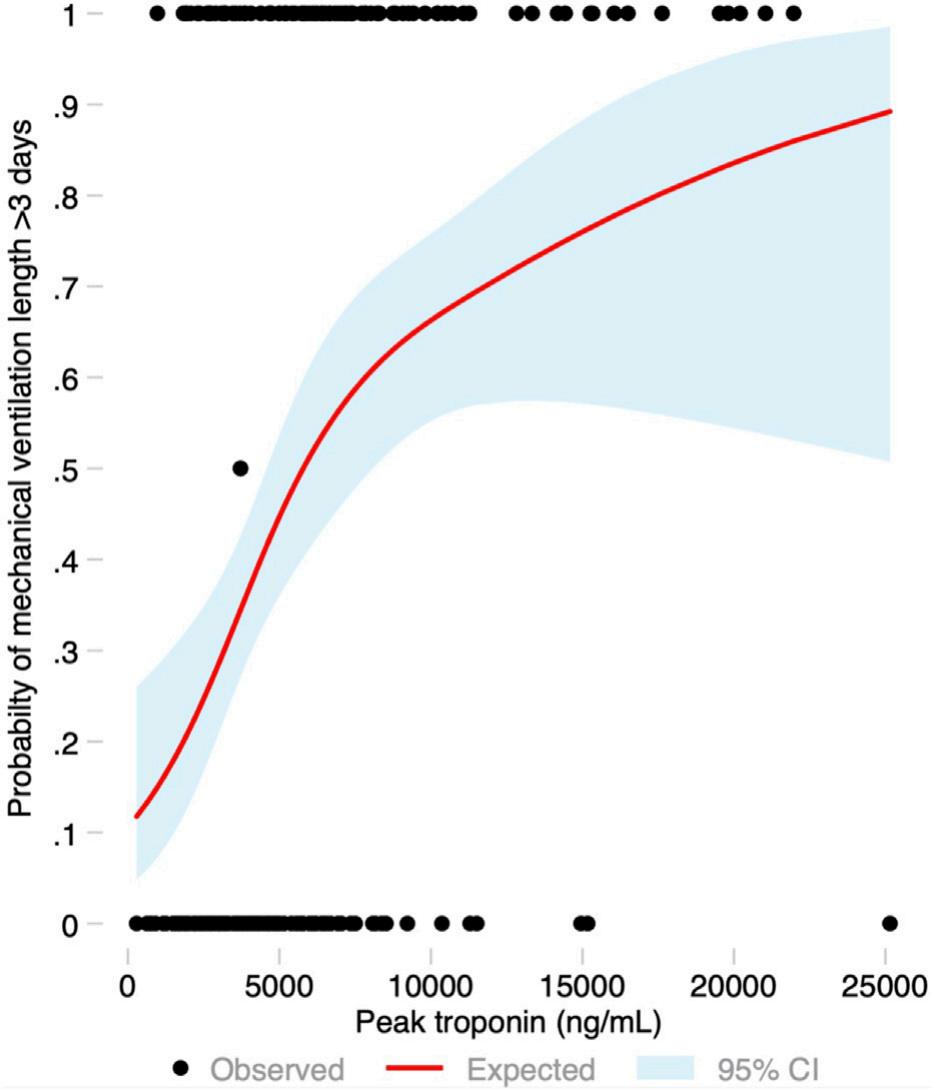
Probability of the primary endpoint (invasive mechanical ventilation for more than 3 days) based on the peak troponin level modeled as a restricted cubic spline, with its 95% confidence interval.

Median ICU stay was 4 (2–14) days, and median hospital stay was 23 (16–53) days. There were 91 (44.2%) patients who met the primary endpoint and were on IMV for >3 days. Peak hs-cTnI was significantly higher in patients that required IMV for >3 days compared to those that were weaned earlier (6,429 vs. 3,823 ng/mL, *p* < 0.001; Figure 3). Peak hs-cTnI alone displayed an AUC = 0.72 (0.65–0.79) to predict prolonged IMV. Peak hs-cTnI was associated with the primary endpoint in both unadjusted (OR per 100 ng/mL increase = 1.01, 95% CI 1.01–1.02) and adjusted analysis (OR per 100 ng/mL increase = 1.02, 95% CI 1.01–1.03). Figure 4 shows how the probability of requiring prolonged IMV increases at higher peak hs-cTnI levels. Table 3 shows univariate predictors for the primary endpoint. Multivariate analysis identified peak hs-cTnI, postoperative VV-ECMO, requirement of more than 1 red blood cell transfusion and pulmonary hypertension as the only independent predictors for prolonged mechanical ventilation.

**TABLE 3:** Odds ratio and 95% confidence interval for the primary endpoint (invasive mechanical ventilation for >3 days) in univariate and multivariate logistic regression analysis.

	Univariate	Multivariate
	Peak troponin	Peak troponin
Hypertension	1.62 (0.80–3.26)	—
Dyslipidemia	1.71 (0.86–3.38)	—
Diabetes	1.95 (0.97–3.92)	—
Chronic heart failure	(collinear)	—
Pulmonary hypertension	2.60 (1.41–4.80)	2.25 (1.05–4.85)
Hemoglobin (per g/L)	0.98 (0.96–0.99)	—
Bridged with VV-ECMO	(collinear)	—
Bridged with VA-ECMO	5.67 (1.55–20.76)	—
Bilateral lung transplant	2.68 (1.26–5.70)	—
Use of EVLP	1.55 (0.86–2.79)	—
CPB during surgery	2.41 (0.78–7.47)	—
VA-ECMO during surgery	2.58 (1.46–4.56)	—
Time on MCS (per min)	1.00 (1.00–1.01)	—
>1 PRBC used	10.19 (4.26–24.40)	7.20 (2.45–21.16)
VV-ECMO after surgery	14.07 (1.77–112.12)	15.26 (1.68–138.51)
Peak troponin (per 100 ng/mL)	1.01 (1.01–1.02)	1.02 (1.01–1.03)

RV, right ventricular; eGFR, estimated glomerular filtration rate; VV-ECMO, veno-venous extracorporeal membrane oxygenation; VA-ECMO, veno-arterial extracorporeal membrane oxygenation; CPB, cardiopulmonary bypass; MCS, mechanical circulatory support; PRBC, packed red blood cells.

The table only shows predictors with a *p*-value <0.2 in univariate analysis. Chronic heart failure (*n* = 1) and preoperative support with VV-ECMO (*n* = 4) were perfect predictors of the endpoint and these patients were therefore excluded from multivariate analysis.

**FIGURE 5:**
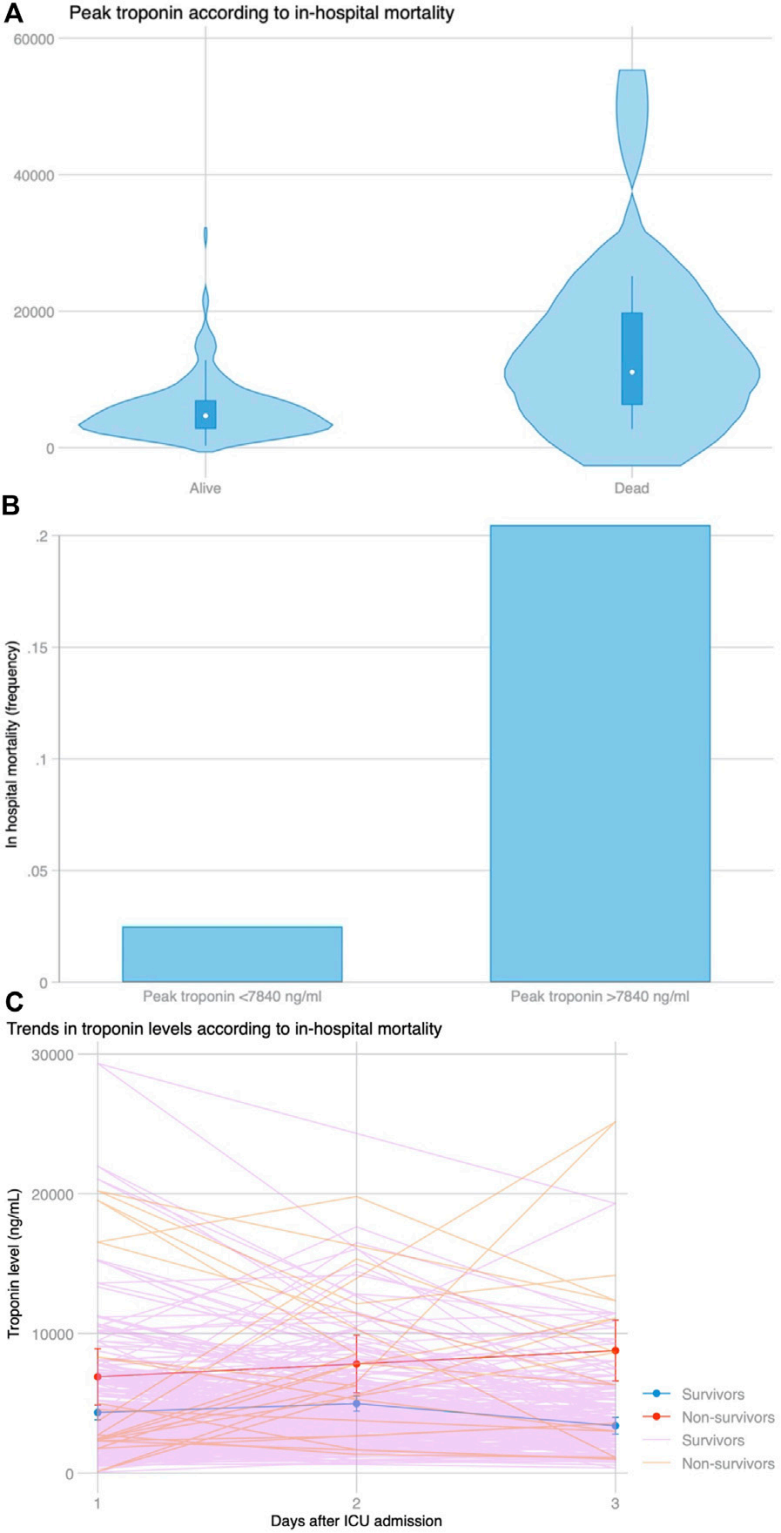
**(A)** Distribution of peak troponin according to in-hospital mortality. **(B)** In-hospital mortality based on the best cut-off for peak troponin (7,840 ng/mL) selected by Youden’s index using ROC curve analysis. **(C)** Trends in troponin levels among patients who survived during admission and those who did not survive the index admission. After a similar increase at day 2 compared to day 1 among both groups (*p* = 0.778), peak troponin continued to rise in patients who died, whereas it declined for patients who survived (*p* = 0.008 for the slope).

There were 13 (6.3%) patients who died during hospital admission. Peak hs-cTnI was significantly higher in patients who died during admission (9,690 vs. 4,659 ng/mL, *p* = 0.001) (Figure 5A). Peak hs-cTnI had an AUC = 0.78 (0.63–0.93) for in-hospital mortality, with the best cut-off at 7,840 ng/mL. Death occurred in 9 (20.5%) patients with a hs-cTnI >7,840 ng/mL and in 4 (2.5%) with a peak hs-cTnI <7,840 ng/mL (*p* < 0.001, Figure 5B). Compared to survivors, patients experiencing in-hospital mortality had a continued hs-cTnI rise with failure to decrease the circulating hs-cTnI levels on the third day (Figure 5C). The association between peak hs-cTnI and mortality remained significant in all bivariate analysis (including MCS use before, during or after surgery, age, diabetes, pulmonary hypertension, or poor RV function).

Atrial arrhythmias were frequent and occurred in 88 (43.4%) patients after LTx. Peak hs-cTnI was associated with the occurrence of atrial arrhythmias (5,833 vs. 4,350 ng/mL, *p* = 0.008) (Table 4). There was no association between peak hs-cTnI and primary graft dysfunction at 72 h after LTx, suggesting that graft dysfunction does not mediate the association between peak hs-cTnI and prolonged IMV. Patients with a peak hs-cTnI above the median had a longer length of stay in ICU (median 8 vs. 3 days, *p* < 0.001), and a longer hospital length of stay (median 29 vs. 21 days, *p* = 0.002). After LTx, only six patients had a coronary angiogram performed as recommended by cardiology consultation based on hs-cTnI trends, and percutaneous coronary intervention was not necessary in any case. There was no correlation between peak hs-cTnI and left ventricular ejection fraction after LTx in those with an available measurement by echocardiography (*n* = 63, Pearson’s r = −0.207, *p* = 0.10).

**TABLE 4:** Peak troponin levels based on the occurrence of the primary and secondary endpoints.

	Number of events	Troponin in those with event	Troponin in those without event	*p*-value
IMV >3 days	91 (44.0%)	6,429 (3,873–9,418)	3,823 (2,392–5,992)	<0.001
In-hospital mortality	13 (6.3%)	11,081 (6,249–19,802)	4,675 (2,773–6,937)	<0.001
Atrial arrhythmias	88 (43.4%)	5,833 (3,334–8,107)	4,350 (2,499–6,428)	0.008
Primary graft dysfunction	48 (33.8%)	4,838 (3,035–7,405)	6,142 (3,749–8,262)	0.087

IMV, invasive mechanical ventilation.

## Discussion

In this study, we measured serial hs-cTnI levels in consecutive LTx recipients upto 72 h after returning to the intensive care setting. We identified that hs-cTnI rise above the defined threshold for MINS was seen in all LTx recipients, and it was associated with perioperative risk factors and not with flow-limiting CAD. After LTx, high peak hs-cTnI is an independent predictor for prolonged IMV, postoperative atrial arrhythmias and in-hospital mortality, probably as a reflection of preoperative and perioperative cardiac stress.

The fourth universal definition of MI defines a type V MI as a CABG-related MI, leaving all other postoperative, non-revascularization related MI within a separate category poorly characterized [12]. Within 72 h after LTx, 99.5% of our population had an elevated hs-cTnI >10 times above the 99th percentile, as documented before [9]. There were no coronary plaque ruptures documented, no significant CAD before surgery, no need for revascularization during hospital admission and no correlation with left ventricular ejection fraction, suggesting that hs-cTnI rise is explained by factors related to the surgical intervention and the postoperative course, especially since the LTx operation necessitates cutting and sewing the atrial myocardium, and the rise in hs-cTnI does not reflect coronary artery disease. The independent predictors observed in our cohort for hs-cTnI rise can be explained by elevated end-diastolic pressures, such as an elevation of RV end-diastolic pressure in those with pre-existent RV dysfunction worsened with pulmonary artery cross-clamp, or elevation in left ventricular end-diastolic pressure with retrograde arterial flow from VA-ECMO. Longer time of surgical manipulation likely explains the association between bilateral LTx with peak hs-cTnI. The association between peak hs-cTnI, length of MCS support and requiring more than one transfusion may also be related to hemodynamic stability and supply-demand ischemia, whereas a worse preoperative eGFR may prevent hs-cTnI washout after LTx. The association with more extensive intreventions, length of cardiac manipulation and comorbidities have also been observed in other non-LT thoracic surgeries [13]. Therefore, our results support that coronary events are rare and peak hs-TnI levels correspond to either type 2 MI due to demand-supply mismatch related to preoperative factors, to the surgical intervention or more likely secondary to direct myocardial injury by cutting and suturing of the recipient and donor myocardium during LTx.

In our cohort, peak hs-cTnI was associated with prolonged IMV, atrial arrhythmias and death. There have been two other studies evaluating the role of hs-cTnI after LTx surgery. In one of them, including 95 patients, higher hs-cTnI measured on arrival to the ICU was found to be associated with mortality, but this was published only as an abstract with no more information being available [8]. Another study described the impact on 30 days and 1-year mortality of a single hs-cTnI measurement upon admission to the ICU. The authors also described an association between a higher troponin and mortality [9]. The association between peak hs-cTnI and worse outcomes is well described in both cardiac and non-cardiac surgery. A recent publication demonstrated a strong association between hs-cTnI elevation and mortality with a similar cutoff to predict death (hs-cTnI >5,670 ng/mL), and a slightly lower cutoff for other complications, as observed in our study [14]. The most likely explanation for this association is that hs-cTnI probably captures several factors that confer a worse prognosis after LTx, as it reflects both direct and indirect cardiac damage, as demonstrated in our study.

Normal troponin kinetics after LTx consist of an early peak at 24–48 h and a progressive decline thereafter, as also noted in a study including only 10 LTx recipients [15]. However, we describe a novel association between failure to decrease hs-cTnI levels and in-hospital death, suggesting that hs-cTnI monitoring up to 72 h may be useful to identify these high-risk patients. We observed that a continued rise in hs-cTnI levels beyond 48 h is of concern, and this trend in hs-cTnI kinetics merits careful review from the clinician to identify alternative diagnoses which may explain the persistent rise in serum levels. If the hs-cTnI levels do not trend down beyond 48 h, this may suggest that hs-cTnI level may not be due to myocardial injury from the surgical intervention alone, although we did not observe in our cohort any significant coronary artery disease that could explain hs-cTnI rise, and the association could be explained by clinical worsening, oxygen supply-demand mismatch or decreased hs-cTnI clearance. A broad differential diagnosis exists and alternative etiologies of persistent hs-cTnI elevation in this population may include cardiac arrhythmias, renal failure, respiratory failure, or sepsis [16].

We must acknowledge the limitations of a single center retrospective study, and conclusions can only be hypothesis-generating. Despite having a relatively low number of deaths, it is the largest analysis of hs-cTnI trends after LTx. Unfortunately, hs-cTnI was not available in all LTx recipients at 48 and 72 h and selection bias cannot be completely excluded, but comparison of patients with missing and non-missing hs-cTnI did not reveal any major differences.

Overall, our study shows that, in LTx recipients, peak hs-cTnI within the initial 72 h after surgery is elevated ten times above the range of MINS in >99% of patients. Peak hs-cTnI is not related to coronary artery disease and is most likely related to surgical manipulation of the cardiac atrial tissue, recipient comorbidities and the clinical situation at the time of LTx, hemodynamic stability during the intervention and perioperative factors. An elevated peak and persistent elevation of hs-cTnI identifies patients with higher rates of prolonged ventilation, atrial arrhythmias, and in-hospital death, as it likely reflects a worsened preoperative status with a greater degree of oxygen supply-demand mismatch during surgery and in the early postoperative period.

## Data Availability

The raw data supporting the conclusion of this article will be made available by the authors, without undue reservation.

## Abbreviations

AUC, area under the curve; IMV, invasive mechanical ventilation; LTx, lung transplant; VV-ECMO, veno-venous extracorporeal membrane oxygenation; BMI, body mass index; CABG, coronary artery bypass graft; CAD, coronary artery disease; CI, confidence interval; COPD, chronc obstructive pulmonary disease; CPB, cardiopulmonary bypass; eGFR, estimated glomerular filtration rate; EVLP, *ex-vivo* lung perfusion; hs-cTnI, high-sensitivity cardiac troponin I; ICU, intensive care unit; MCS, mechanical circulatory support; MI, myocardial infarction; OR, odds ratio; PCI, percutaneous coronary intervention; PGD, primary graft dysfunction; ROC, receiver-operating characteristic; RV, right ventricular; VA-ECMO, veno-arterial extracorporeal membrane oxygenation.
